# Collaborative learning of new information in older age: a systematic review

**DOI:** 10.1098/rsos.211595

**Published:** 2023-10-04

**Authors:** Kelly Wolfe, Catherine J. Crompton, Paul Hoffman, Sarah E. MacPherson

**Affiliations:** ^1^ Psychology, School of Philosophy, Psychology and Language Sciences, University of Edinburgh, Edinburgh, UK; ^2^ Division of Psychiatry, Centre for Clinical Brain Sciences, University of Edinburgh, Edinburgh, UK

**Keywords:** learning, collaborative learning, older age, memory

## Abstract

Ageing is accompanied by a multitude of changes in cognitive abilities, which in turn affect learning. Learning collaboratively may benefit older adults by negating some of these age-related changes. However, studies on collaborative learning in older age differ in their methodology and findings. This systematic review provides an overview of the current research on collaborative learning in older age, exploring what factors influence collaborative learning in this age group. The titles and abstracts of imported 6629 works were screened, as well as four works added manually, which resulted in 29 studies. These studies were conducted across five countries (Canada, United States, United Kingdom, Switzerland and Belgium) between 1993 and 2023. Most studies were quantitative with a non-randomized (*n* = 16) design. Of the 29 studies, almost all studied collaboration in pairs (*n* = 28). The results suggest that the benefits of collaborating in older age may depend on the type of learning material, that familiarity between partners does not affect learning, and that age differences appear to decrease or disappear when older adults are provided with adequate time or trials. In addition, this systematic review identifies several gaps in the literature that future research should investigate further. This study was preregistered prior to its commencement on 21 January 2022. The accepted Stage 1 manuscript, unchanged from the point of in-principle acceptance, may be viewed at https://osf.io/tj4w7/. The data and materials of this study can be found at https://osf.io/8xvqf/.

## 1. Introduction

Generally, society holds conflicting beliefs about older age; with older age comes wisdom, but also a decline in cognitive functioning. Though seemingly contrary, research has found that both beliefs can coexist; older adults are found to experience more emotional stability [1], show signs of increased positivity [2,3], and yet experience declines in cognitive functions such as processing speed, executive functions and memory [4,5]. Those cognitive abilities are associated with the ability to learn new information and skills, and age-related decline can result in older adults experiencing learning difficulties [6]. In general, learning is studied as a solitary process, in which a person's ability to learn is measured in an experimental setting, using a task with specific instructions on which information or skill to learn. Yet, in daily life, people often learn through interacting with others, or by working collaboratively on obtaining knowledge. Learning collaboratively may be especially beneficial in older age as it could negate age-related effects on learning. For example, findings by Derksen *et al*. [7] showed that older adults performed similarly to younger adults on a memory task when learning collaboratively. As such, it is advantageous to investigate the degree to which collaborative learning benefits older adults, as well as the circumstances under which this occurs.

### 1.1. Age-related effects on abilities associated with learning

The ability to learn requires multiple cognitive processes including encoding, maintaining and retrieving knowledge, some of which are affected by age. Even though age-related effects on memory, reasoning, and speed appear most pronounced after the sixth decade [8,9], some age-related effects can be found in middle age [10] or even earlier [8]. In addition, the age-related effects on cognitive abilities such as processing speed and working memory can be substantial [11]. Processing speed has an indirect effect on learning in older age; being older is related to lower processing speed, which is then related to lower accuracy when learning new information, which in turn is associated with higher rates of forgetting [12]. Processing speed has been found to affect the relationship between older age and multiple cognitive processes [11–15], including working memory [16]. Working memory is the cognitive system responsible for temporarily holding information and using it for the execution of cognitive tasks, and is related to information processing, comprehension, problem-solving, and learning [17]. Prior research has shown that older adults are likely to experience a decline in working memory [11,18–22], with Elliott *et al*. [19] reporting that older adults experienced increased difficulty across all working memory tasks in their study. A decline in working memory can affect performance on a learning task, such as not being able to remember instructions [23], or not being able to assess relationships between pieces of information or create new associations. These difficulties may manifest due to an impairment in the encoding, maintenance or retrieval stage of working memory [24].

### 1.2. Negating age-related effects on memory

However, age-related effects on memory can be reduced under certain conditions. If the information is emotional by nature, older adults remembered a similar amount of information to younger adults [25,26]. For example, Comblain *et al*. [27] found that older and younger adults performed similarly when recognizing emotional information, with older adults recognizing stimuli due to their own emotional response when the information was presented to them at the time. Another approach to reducing age-related effects on memory is through inducing mood [2]. Grady *et al*. [28] found that older adults recognized more positive and negative faces when they were in a positive mood, while Knight *et al*. [29] found that older adults remembered sad information better when a negative mood was induced. In addition, when non-declarative memory is involved, older adults retain a similar amount of knowledge to younger adults when learning a skill [30,31]. Lastly, working collaboratively in a social setting could negate age-related effects on memory. Existing research has highlighted the relationship between social engagement and cognitive well-being, with low social engagement associated with a higher likelihood of cognitive decline [32–34]. Some studies have found evidence that age-related effects on memory can be negated through collaboration [35–37].

### 1.3. Collaborative recall

We often collaborate with others to remember an event or experience and many studies have investigated remembering, or recall, as a collaborative experience. Typically, in such studies, participants cooperate to recall information that they obtained previously, either individually or as part of a pair or larger group. These pairs or groups can consist of strangers [38–43], friends [38,39,42–44], siblings [42] or romantic partners [41,45–47]. The information to be recalled differs, and study materials range from a list of words without personal relevance [44,46,47], actions within a computerized memory task [41], video materials [38,40,43], a list with items of personal relevance [46], stories [38,47] and autobiographical interviews [46]. So far, research on collaborative recall has demonstrated that remembering together can be highly beneficial compared to remembering individually [40,43,48,49]. Collaborative recall has been found to increase the accuracy and completeness of a memory compared to working alone [50] and its success was found to be stable over time [45].

Recalling collaboratively may be especially beneficial in older age, with studies showing that older adults remember more after recalling together compared to recalling alone [47,51–53]. In addition, the benefits of the collaborative recall over individual recall were found to still be present 7 days later, in both younger and older adults [52]. The benefits associated with remembering together are mostly found in intimate pairs such as spouses [41], but the extent of success depends on factors beyond the type of relationship. Differences in the division of labour [47], the strategies used to recall, and effective communication skills [46] can impact the extent to which collaborative recall is beneficial. Overall, the research on collaborative recall has expanded over the last two decades, including several reviews (e.g. [51,54,55]), and has demonstrated that remembering together can provide benefits in older age. If collaboration benefits older adults when they recall existing information, could it also help when they are learning new information?

### 1.4. Collaborative learning

Collaborative learning can be defined as cooperating and communicating with another person to learn a new skill or task, as opposed to learning alone [56,57]. In collaborative learning studies, cooperation occurs during the acquisition of new information or skills, rather than only during their retrieval (which applies to collaborative recall). Learning collaboratively can be done in a variety of ways. People may learn with a person familiar to them, such as a spouse or friend, or with an unfamiliar person. Pairs, as well as larger groups of more than 2 people, can work together to acquire new skills or information. Within these types of groups, there may be an equal (i.e. both work on the same task with the same roles) or unequal division of labour (i.e. pairs have defined but different roles within a collaborative activity). In addition, the learning process can be face-to-face or through other media, such as online or over the telephone [56]. Like collaborative recall, a variety of materials can be used to study how people learn together. The Barrier Task [58] is such a material that has been used in collaborative learning research [7]. This joint referential task is performed by two people, who are assigned the role of Director or Matcher. They sit facing each other across a table while separated by a low barrier, which obstructs the view of the other side of the table. Each person has a board with twelve numbered spaces and twelve tangrams (which are abstract geometric shapes). The Director's tangrams are arranged in a predetermined order on their board, while those of the Matcher are not. The Director must communicate to the Matcher where the tangrams are to be placed to have two identical boards by the end of the trial. This process is completed by describing what each tangram looks like and where to place them on the board. Over time, participants learn how to complete the task more efficiently through their own acquired knowledge of the task as well as the complementary knowledge of their collaborator (i.e. utilizing the other person's labels and descriptions of the tangrams). As such, the Barrier Task is an example that clearly demonstrates how collaboration between pairs can result in new knowledge or skills.

By learning collaboratively, participants can use their prior knowledge, which can be shared or complementary. Shared knowledge refers to group members having (close to) identical prior knowledge of the same topic, whereas complementary knowledge means that each collaborator provides individual knowledge of a shared topic [59].

There are two mechanisms that underlie both collaborative learning and recall, namely cross-cueing and re-exposure. In cross-cueing, the recall of one group member can cue the memory of other group members [60]. For example, expert pilots recalling aviation scenarios together remembered more compared to the scenarios being remembered individually [61]. Secondly, re-exposure to information occurs when one person provides access to learning material that would not have been available if pairs learned alone. For example, one person may not remember what they ate at a family meal the week before, but someone else present at that same dinner may remember. When information is remembered together, a person is re-exposed to the information due to their partner's recall and is more likely to remember what they had for dinner than if they remembered alone [51].

Though there is evidence that people learn better together, some studies suggest that the collectively learned information is unlikely to be equal to the pooled performance of individual group members when remembering collaboratively [62]. Pereira-Pasarin & Rajaram [63] compared a collaborative group to a nominal group (the same number of participants remembering individually) and found that participants did not contribute as much information when learning with a partner compared to learning alone. This effect is referred to as collaborative inhibition [49,64]. Collaborative inhibition likely presents due to varying retrieval strategies across participants [65], and a lack of cross-cueing. Group members remembering collectively often do not cue each other to elicit new information, beyond what has been mentioned previously [48,55]. However, the effect of collaborative inhibition disappears when partners can work together to remember (unlike pairs recalling while together, but without any conversation between partners) [66]. In addition, collaborative inhibition has not been found when recalling semantic information; when recalling semantic information, pairs perform better together compared to individual recall [47]. In addition, pairs have been found to recall more accurate and complete information compared to individual recall [50]. Overall, the benefit of collaborative learning may depend on other factors, such as the type of information and how pairs work together (e.g. learning together but remembering alone or being allowed to help the other person remember).

While children and younger adults are known to learn collaboratively (both in and outside school environments) [67], collaborative learning may be equally prominent in older age as older adults often work together with a spouse, friend or family member in everyday cognitive activities [53,68,69] and generally view collaboration as beneficial [70]. These regular experiences of working collaboratively allow for individual strengths to be applied where needed and more efficient use of the cognitive resources that are available, especially during tasks without much guidance or structure [7]. As such, collaborative learning may benefit older adults' memory performance when learning new information.

In the last two decades or so, research on collaborative learning has experienced a resurgence. For example, studies on collaborative learning among patients with cognitive impairment have found that learning together improved memory performance, in both patients with Alzheimer's disease [71] and patients with amnesia due to focal hippocampal damage [58]. These findings highlight the potential benefits of learning together, and how this social approach to learning may be able to negate memory decline in older age.

### 1.5. The distinction between collaborative learning and recall

Though collaborative learning and recall are often used interchangeably, as well as studied simultaneously, here we make a distinction between the two concepts. Collaborative recall specifically refers to the process of solely retrieving information, while collaborative learning encompasses multiple processes, including understanding, processing, and storing information, as well as being able to recall information with other people. While collaborative recall can be a part of the learning process, it does not have to be, and these two areas can be studied separately (i.e. many studies on recall do not involve learning new information or skills). Collaborative recall has been studied extensively in past years (for reviews, see [51,54,55]) and has shown benefits for older adults. However, it is not clear whether the same benefits can be found in collaborative learning, and if the mechanisms that make collaborative recall successful are present when learning collaboratively. As such, a review of the literature on collaborative learning may be overdue.

### 1.6. Present research

A systematic review of the collaborative learning literature specific to older people is timely and important for several reasons. Firstly, from a societal perspective, Western societies are ageing, and one in four people is predicted to be aged 65 years or older in the United Kingdom in 2050 [72]. Because of this increase in population age, age-related cognitive decline will affect more people than ever before. Collaborative learning may offer benefits that can help negate these age-related effects on cognition. In addition, with the rise in population age, people will be required to work longer until they are able to retire. Being able to learn new skills and information is essential to many professions, and as such, research on how to best support older adults in their learning process would benefit both the individual and the workforce.

Secondly, most of the literature has investigated the effects of collaboration on recall, and it is currently unclear whether the same factors are important for collaboration during the acquisition of knowledge. Whereas recall has been well-studied, and reviews on collaborative recall in older age are already available (see [51,54,55]), as well as a systematic review on preparing for collaborative learning [73], there is currently no review on collaborative learning in older age.

Studies on collaborative learning in older age have been scarce, but research on this area has experienced a resurgence in the last two or so decades. However, the existing research on this topic has used a range of methodologies, analyses, and populations and findings are mixed. As this field of research is expanding, a systematic review of the research on collaborative learning can provide a clear, concise overview of the current methods, analyses and findings. In addition, the proposed systematic review can also identify potential gaps and opportunities for future research to address.

To conclude, we conducted a systematic review on collaborative learning in older age to assess the benefits of learning together in negating age-related effects. As studies on collaborative learning in older age are limited and use different designs (i.e. using married couples or younger versus older adults, as well as computerized versus on-paper materials), this systematic analysis focused on providing a narrative review of the research conducted so far, as well as identifying gaps in the current literature. The systematic review includes collaborative learning of new information only, excluding autobiographical (i.e. personal memories) and prior-known semantic information, and explores two research questions: what research has been conducted so far on collaborative learning in older age, and under what circumstances is learning collaboratively more beneficial than learning individually?

## 2. Method

### 2.1. Literature search

We used the following methods to locate articles examining the topic of collaborative learning in older age:

1. A computerized search on Web of Science, PubMed, and Google Scholar for articles on collaborative learning in older age groups^1^. The following keywords were used (for all searchable fields, such as title, abstract, and keywords): collaborat* learn* OR collaborat* memor* OR "joint referential task" OR "barrier task" AND older age OR older adult. Specific to Google Scholar, we only included the first 5 pages of results^2^. For Prospero and EThOS, we used fewer keywords as these databases do not accept the use of asterisks: collaborative and older. For an overview of all search terms, see table 1.
2. A computerized search in PhD thesis libraries, including EThOS and EBSCO Open Dissertations.
3. A manual search by emailing authors to enquire about existing work that has not been published (yet), such as PhD theses or preprints. In addition, we also contacted authors if search results include an abstract or poster that does not have sufficient information to be included as it is.
4. A computerized review of articles cited in papers found in the initial search (1), to determine they fit the inclusion criteria. This search was only conducted on works that had been selected for inclusion after full-text review.
5. A computerized review of articles that cited the papers found in the initial search (1) that discussed any of the keywords listed above. This search was only conducted on works that were selected for inclusion after full-text review.

**Table 1.** An overview of search terms for each database.

database or search engine	search terms	type of search
Web of Science	(collaborat* learn* OR collaborat* memor* OR "joint referential task" OR "barrier task") AND (older age OR older adult)	all fields
PubMed	(collaborat* learn* OR collaborat* memor* OR "joint referential task" OR "barrier task") AND (older age OR older adult)	all fields
Google Scholar	(collaborat* learn* OR collaborat* memor* OR "joint referential task" OR "barrier task") AND (older age OR older adult)	all fields
PsychInfo	(collaborat* learn* OR collaborat* memor* OR "joint referential task" OR "barrier task") AND (older age OR older adult)	all fields
Prospero	collaborative learning^3^	all fields
EBSCO	(collaborat* learn* OR collaborat* memor* OR "joint referential task" OR "barrier task") AND (older age OR older adult)	all fields
EThOS	collaborative learning	all fields

The search terms listed in table 1 are a combination of key characteristics that each relevant work was required to have and a common type of material that had been used in prior studies on collaborative learning in older age. These terms were not selected through iterative scoping searches. Instead, we selected the search terms initially and then conducted scoping searches to see whether these terms would result in relevant works.

### 2.2. Inclusion criteria

1. Works that examined collaborative learning, defined as two or more individuals interacting to jointly acquire new information or a new skill. A distinction was made between collaborative recall and learning; studies examining collaboration only during recall, not as part of collaborative learning, were excluded.
2. Works that included an older adult population. If studies included two or more populations, one of which are older adults, we only included information relating to the older adult group (if information regarding the older adult group was clearly separate from any other participant group).
3. Works that included a healthy population (of older adults). If studies included two or more populations, one of which was healthy older adults, we only included information relating to the healthy older adult group (if the information relating to the healthy sample was available separately).
4. Works that reported a healthy older adult sample with a mean age of 60 years or older.
5. Works that provided participants with new information to learn. Some studies used autobiographical information, which is difficult to verify in terms of how much is remembered or whether the memory is correct, as well as existing semantic information, such as names of US presidents or cities. This type of semantic information is often general knowledge and is known before the commencement of the study. If a work used both new and existing information and has distinct conditions, we included it and extracted the work on new information only.
6. Types of works included were reviews (e.g. meta-analysis, mini-review, systematic review), research articles, conference proceedings or abstracts, posters, PhD theses and preprints. We also included book chapters to locate any relevant works discussed in the chapter.
7. Any works that included the listed criteria above and were written in English.

### 2.3. Exclusion criteria

1. Works that did not focus on collaborative learning. This also excluded works with a focus only on collaborative recall.
2. Works that did not include an older adult sample.
3. Works that did not include a separate healthy older adult sample (this age group had to be clearly distinguishable from other groups).
4. Works with samples of which the mean age was lower than 60 years or did not clearly state the mean age of the sample.
5. Works that used existing semantic information or personal (i.e. autobiographical) information.
6. Works that were deemed an inappropriate format for this review (see inclusion criterion 6).
7. Works that were not written in English.
8. Works that did not clearly reference or discuss the materials used in the study/studies.
9. Works that did not clearly mention the type of design that had been used (i.e. a between or within groups design).
10. Works that did not include the statistical outcome(s) or other forms of analysis (some work may be qualitative), or statistical outcomes that could not be interpreted without additional information. For example, a poster or abstract must mention what type of statistical test was used and include the test value, degrees of freedom, and the associated *p*-value (if frequentist, similar requirements apply for Bayesian analyses).

### 2.4. Procedure

#### 2.4.1. Literature research

We started our literature search by using the listed keywords to search multiple databases for articles relating to collaborative learning in older age. Our search also provided conference abstracts, for which we contacted the authors to enquire whether any more extensive (unpublished) works were available. In addition, authors whose works were included in the review were contacted for possible unpublished materials. As the systematic review was not completed within 12 months, we used the same search criteria to conduct an additional literature search for works that were published after our initial search (8 March 2022). All works included in the title and abstract screening phase are available to download and read into a reference manager, and all manual searches are available for replication purposes.

#### 2.4.2. Article screening

Following our literature search, references were imported into Covidence, and we proceeded by screening titles and abstracts. Any works selected at this stage were subjected to a full-text review (step 3). A second reviewer independently screened 10% of the articles at both stages to ensure that inclusion criteria were sound. Any initial disagreements on inclusion of the 10% of works screened by two reviewers were resolved by a discussion between the primary reviewer (who screened 100% of works) and the secondary reviewer (who screened 10% of works). In the case of a discussion between the primary and secondary reviewer not leading to a joint decision, the remaining two co-authors would provide their decision on whether that work was or was not included in the systematic review, but this was not required. The decisions made by all involved were blinded.

#### 2.4.3. Interpreting selected works

Lastly, the included works were thoroughly compared and contrasted with other included works in terms of their methods, area of research within collaborative learning in older age (i.e. participants who are spouses, unfamiliar or young versus older adults), and results (step 4), which can be found in the results section below. The current paper aims to provide the reader with a comprehensive overview of differences between studies that use a similar design (i.e. all using familiar partners as a learning partner), as well as differences between study designs overall (i.e. learning with a familiar or unfamiliar partner), resulting in detailed knowledge of the work done on collaborative learning in older age thus far.

#### 2.4.4. Further information

To develop the method of this systematic review, we used the Non-Intervention, Reproducible and Open Systematic Reviews (NIRO-SR) framework [77]. In addition, PRISMA guidelines were used to ensure transparent reporting. Copies of the completed NIRO-SR and PRISMA documents are available on the project's Open Science Framework page, at https://osf.io/8xvqf/.

## 3. Results

**Figure 1.**
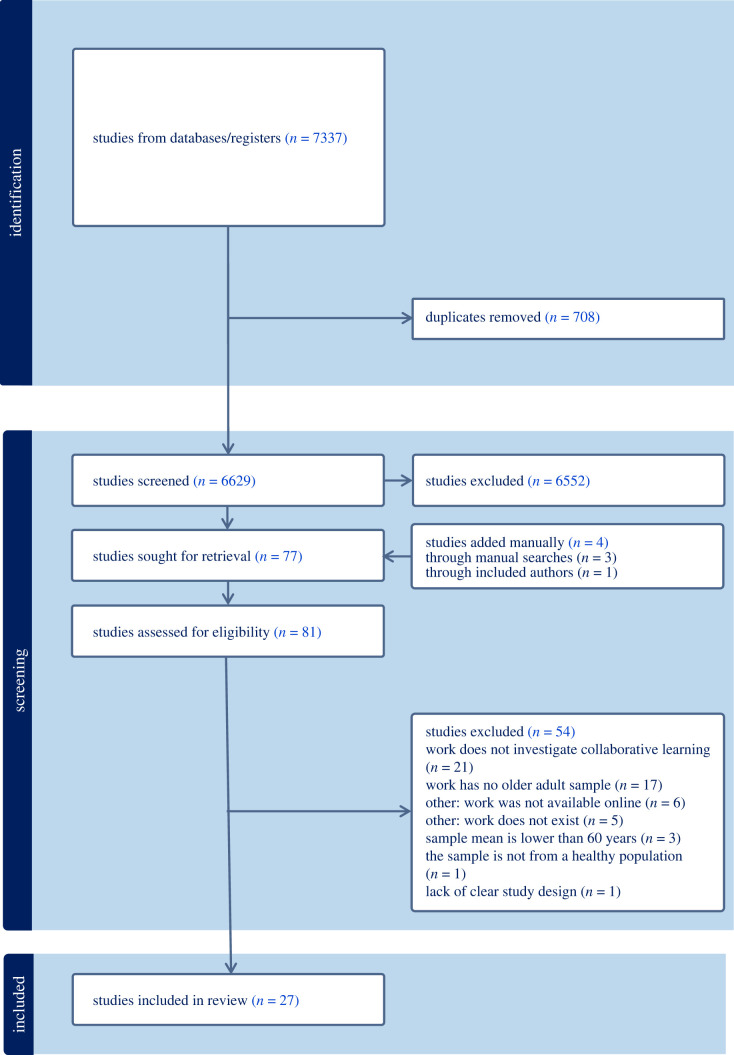
PRISMA chart of the systematic review conducted in Covidence.

We screened the titles and abstracts of 6629 works, assessed the full text of 77 articles and found 23 articles that met all inclusion criteria (figure 1) through our pre-registered searches. In addition, four additional works were identified through manual searches and through contact with the authors [78–81], resulting in 27 works. These 27 works were conducted across 5 countries (Canada, United States, United Kingdom, Switzerland, and Belgium) between 1993 and 2023, spanning 30 years. Of these 27 works, two works included multiple studies [82,83]. As such, a total of 29 studies were included in this review.

In terms of study design, most studies investigating collaborative learning were quantitative studies with a non-randomized (*n* = 16) or randomized (*n* = 9) design. Only three studies had a fully qualitative design (*n* = 3) and one study had a mixed design (*n* = 1). Of the 29 studies, almost all studied collaboration in pairs (*n* = 28), and one study studied collaboration in a group larger than 2 people (*n* = 1). We used study characteristics to create categories of studies on collaborative learning, which can be found below. Each category discusses studies that incorporate that specific aspect in their research. However, as some studies may include multiple design features, some studies are discussed in more than one section. For an overview of studies, their materials and outcomes, see table 2.

**Table 2.** Overview of included works. NIH, National Institutes of Health; IT, information technology; GPS, Global Positioning System.

author(s)	country	article type	type of research	total sample size	compared with young adults	compared with learning alone	relationship with co-learners	materials	outcomes
Cheng & Strough [84]	United States	research article	randomized experimental study^a^	97	Y	Y	familiar	everyday problem-solving task	pairs were more accurate than individuals. Younger adults were faster and more accurate than older adults
Crompton [82]	United Kingdom	PhD thesis	non-randomized experimental study	48	Y	N	familiar, unfamiliar	adapted version of Map Task	no effect of age group or familiarity. Younger adults did have improved delayed recall
	United Kingdom	PhD thesis	non-randomized experimental study	24	N	N	perceived human versus computer partner	adapted version of Map Task	older adults modified their interaction when learning with a human or a computer. Older adults recalled fewer details of routes learned with the computer partner and found the learning experience more difficult
Crompton & MacPherson [85]	United Kingdom	research article	non-randomized experimental study	24	N	N	perceived human versus computer partner	referential task/Barrier Task	participants in the computer condition were slower, took fewer turns, and recalled less
Crompton *et al*. [86]	United Kingdom	research article	non-randomized experimental study	48	Y	N	familiar, unfamiliar	referential task/Barrier Task	older adults’ performance became indistinguishable from younger adults over time, no effect of familiarity
Derksen *et al*. [7]	United States	research article	non-randomized experimental study	68	Y	N	familiar	referential task/Barrier Task	older adults' performance became indistinguishable from younger adults over time
Filer & Scukanec [87]	United States	research article	non-randomized experimental study	8	Y	N	unfamiliar	referential task/Barrier Task	younger women used fewer words, turns, and had fewer errors than older adult women
Gould *et al*. [88]	United States	research article	non-randomized experimental study	60	Y	N	familiar, unfamiliar	referential task/Barrier Task	younger adults performed better overall (including number of words, turns, and optimal descriptions) compared to older adults, with no effect of familiarity
Hupet *et al*. [89]	Belgium	research article	non-randomized experimental study	40	Y	N	unfamiliar	referential task/Barrier Task	older adults’ performance improved but not to the extent that younger adults' performance did
Kannampallil *et al*. [81]	United States	research article	randomized experimental study	144	N	N	unfamiliar	medication schedulers	older adults created more accurate medication schedules when using a structured aid, with no difference between structured and unstructured aids when considering medication complexity
Margrett *et al*. [90]	United States	research article	non-randomized experimental study	22	Y	N	familiar	adapted version of VW task	couples actively worked together, most often tutoring each other. Middle-aged adults outperformed older adults
Margrett & Willis [91]	United States	research article	randomized experimental study	98	N	Y	familiar	multi-week training on inductive reasoning strategies	receiving training is generally beneficial, there was no difference in accuracy between working alone and working together
Morrow *et al*. [83]	United States	research article	randomized experimental study	96	N	N	unfamiliar	medication scheduler	aids are associated with higher accuracy, especially when the medical scenario is complex, compared to an unstructured aid (pen and paper) or no aid
	United States	research article	randomized experimental study	64	N	N	unfamiliar	medication scheduler	participants were more accurate when using the medication scheduler, but there was no difference in speed
Peter-Wight & Martin [53]	Switzerland	research article	non-randomized experimental study	141	N	Y	familiar	Black Box task	participants' performance was better when working collaboratively versus working alone
Rodrigues *et al*. [92]	United States	research article	non-randomized experimental study	32	Y	N	familiar, unfamiliar	referential task/Barrier Task	participants became more efficient over time, regardless of age and familiarity. Younger adults were more efficient in communicating with a familiar partner, older adults were more efficient in communicating with an unfamiliar partner
Seah *et al*. [79]	Canada	research article	non-randomized mixed method study (quantitative and qualitative)	50	N	N	largely unfamiliar	bingo nutrition and health game	participants significantly improved their knowledge of health and nutrition, had a more positive attitude towards digital games, and their social connectedness increased. Common themes in the interviews consisted of social connectedness and knowledge of technology, health and nutrition
Saczynski *et al*. [93]	United States	research article	randomized experimental study	96	N	Y	familiar	multi-week training on inductive reasoning strategies	individual and collaborative training groups did not differ in performance, except when both completing a collaborative recall test (in which individuals working together did worse)
Sayago *et al*. [78]	United Kingdom, Spain	research article	ethnographical study with semi-structured interviews	420	N	N	largely unfamiliar^b^	multi-week training on basic computing skills	during initial learning, participants mostly worked with the same partner. Participants found interacting with unfamiliar peers difficult due to different strategies or strategies had to be created and agreed
Vazquez *et al*. [94]	United States	research article	randomized experimental study	466	N	Y	largely unfamiliar^b^	tutorial on searching for health information online (NIH)	no effect of collaboration in terms of satisfaction or performance. Previous IT experience may mediate the relationship between learning outcomes and learning methods
Vrkljan [95]	Canada	research article	non-randomized experimental study	44	N	N	familiar	using a GPS system	couples less experienced with technology had higher levels of assistance, mostly supportive. Couples more experienced with technology provided less assistance but the assistance that they did provide was more directive
Vrkljan [96]	Canada	research article	non-randomized experimental study	40	N	N	familiar	using a GPS system	couples more experienced with technology made fewer errors overall, performed each task at a faster rate, and provided less assistance
Wei *et al*. [97]	Canada	research article	qualitative research	22	N	N	familiar	semi-structured interview and open-ended questions	older adults often search online for daily tasks, interests, and curiosity. The latter is often to settle a disagreement or to discuss a topic together
Wood *et al*. [80]	Canada	research article	non-randomized experimental study	64	N	N	largely familiar^b^	training sessions to develop computing skills	collaborations were considered helpful and effective, and participants were more involved
Xie [103]	United States	research article	randomized experimental study	124	N	Y	largely unfamiliar^b^	tutorial on evaluating Internet health information (NIH)	performance improved regardless of condition or familiarity
Xie [104]	United States	research article	non-randomized experimental study	172	N	N	familiar, unfamiliar^b^	tutorial on searching for health information online (NIH)	performance improved regardless of familiarity
Xie [105]	United States	research article	randomized experimental study	146	N	Y	largely unfamiliar^b^	tutorial on helping older adults search for health information online (NIH)	performance improved regardless of collaboration or learning design
Yoon & Stine-Morrow [98]	United States	research article	non-randomized experimental study	48	Y	N	unfamiliar	referential task	older adults found it more difficult to identify the source of a description and adjusted their descriptions less when working with a naïve partner compared to younger adults
Zhang *et al*. [99]	Canada	research article	observational study	22	N	N	unfamiliar^c^	Wii Sports Resort games	older adults learn through questions and feedback of their younger partners, such as pointing out errors or explaining concepts

^a^Indicates studies that are largely randomized but have made minor changes or specifications that were not random.

^b^Indicates study designs that were largely unfamiliar or unfamiliar, but also had some participants included that did not fit this design.

^c^Both younger and older adults were involved in this study. However, the younger adults were involved as a guide and their learning performance was not assessed. As such, this study is not included in the younger versus older category.

### 3.1. Materials used to study collaborative learning

Across the included works, a range of different materials were used to study collaborative learning. Some were computerized tasks with specific measurements of success rate, whereas others were more subjective or aimed at participants' perspectives of the success of learning together. This section discusses the most common types of materials used to study collaborative learning.

#### 3.1.1. Referential task

The most common material in collaborative learning studies (*n* = 8) were variations of a collaborative referencing task, referred to as the Barrier Task in some studies [7,86,87]. In this task, pairs collaborate to create two identical grids of abstract images, while being separated by a barrier. One learner (the director) describes the order of the images in their grid and the other (the matcher) attempts to recreate the same order in their own grid. Task performance improves over time, as pairs learn to communicate the images and their locations to each other more efficiently. Generally, performance on this task is measured through the number of correctly placed images on the grid, the number of conversational turns taken between pairs, the number of words spoken by each person or combined, and the time it took pairs to complete each grid.

However, studies differed in their design or execution of the task, as there were variations in several aspects of the task, including the number of images used. This is likely due to the different versions available, including those produced by Clark & Wilkes-Gibbs [100] and Krauss & Weinheimer [101]. Some studies used 10 images per trial [88,89] while others used 12 images [7,85,86,92,98]. Also, the number of trials per session differed, with 4 trials [88,98], 6 trials [7,87,89], 9 trials [85,86] or more [92] per condition. Lastly, some pairs completed the task via a computer [86,98], whereas others did it in person.

The task has been used to study different settings of collaborative learning. For example, when used to study familiar versus unfamiliar partners, Crompton *et al*. [86] and Gould *et al*. [88] found no differences in task performance (i.e. number of turns, words and time to complete), while Rodrigues *et al*. [92] found that older adults were more efficient communicating with an unfamiliar partner as they used fewer words to explain the images. When using the referential task to compare the performance of younger and older adults, varying effects have been found, including no age differences [98], older adults being initially less efficient than younger adults but this difference decreasing over time [7,86,89], or mixed findings as the age difference was dependent on whether pairs were familiar or unfamiliar [92].

#### 3.1.2. Map Task

Two studies used an adapted version of the Map Task [102]. In this task, participants are asked to work collaboratively to recreate a route on a map, with each partner only seeing half of the intended route. Through conversation, pairs must establish the correct route from start to finish, as they are separated by a barrier. Generally, performance on the Map Task is measured through the accuracy of the route drawn by participants (with a maximum score of 18), as well as the number of words used, number of turns taken, and time to complete the trial. One of the two studies using the Map Task examined familiar versus unfamiliar pairs and differences between younger and older adults [82]. It found no differences between familiar and unfamiliar pairs, nor did older and younger adults differ in their performance (i.e. accuracy of the route, the number of words and turns, and time to complete the trial) except for the delayed recall of the routes a week post-test, with younger adults remembering the routes better than older adults. The second study by Crompton [82] explored human–computer interactions, and whether collaboration would differ when older adults perceived their partner to be a human or a computer. Participants became more efficient over time (i.e. less time to complete the route, more accurate and fewer words and turns used), regardless of who they perceived their partner was, but recalled more of the routes that they had completed with a (perceived) human instead of a computer [82].

#### 3.1.3. Using a GPS system

Two studies by Vrkljan [95,96] used a GPS system to study learning together. The first study had both a younger and older adult sample. Results showed that older adults were slower than younger adults in planning their routes and made more mistakes. The study also found that prior experience with technology affected participants’ performance on the GPS system, with older adults having significantly less experience with technology compared to younger adults as well as lower learning performance. In the second study, the effect of technology was assessed with just older adult participants, but no effect of experience on learning performance was found. It is possible that this effect is largely age-dependent, as there could be insufficient variation in GPS experience within an older adult sample.

#### 3.1.4. Training in computer skills

In several works, collaborative learning was studied using an informal setting in which participants gained new skills or information with others. For example, several studies used pre-existing courses on computing skills to examine older adults' learning with another person. For example, studies by Xie [103–105] and Vazquez *et al*. [94] used tutorials on helping older adults search for health information online and evaluating Internet health information, created and distributed by the National Institutes of Health (NIH). Across these four studies using these tutorials, participants’ performance improved regardless of whether they collaborated with another person or worked by themselves. However, when taking prior computing experience into account, Vazquez *et al*. [94] found that those with more computer experience showed more long-term learning in the collaborative condition, whereas those with less computer experience had more long-term learning when working alone.

Wood *et al*. [80] and Sayago *et al*. [78] also used training sessions to develop older adults' computing skills. In the study by Wood *et al*. [80], older adults were encouraged to work together but could choose to work alone. The older adults in the study reported that they preferred learning with a partner, as they found collaborating helpful and effective. In the study by Sayago *et al*. [78], participants could decide who to work with and could switch between partners at any time. They found that older adults preferred working with someone they had got to know earlier in the study compared to an unfamiliar partner. They reported that they often had a pre-existing shared strategy with their known partner (not to be mistaken with familiar, which encompasses a more long-term relationship beyond the study), while unfamiliar partners often used learning strategies different from their own.

#### 3.1.5. Training in reasoning skills

Studies also used multi-week training sessions to examine how participants learned inductive reasoning strategies [91,93]. Participants were sent materials to complete together or alone each week and were asked to come in for a pre- and post-test. Examining between conditions, these two studies found that training was more beneficial than no training, but there was no difference in working individually or with a familiar partner [91,93]. However, when comparing on an individual level, participants in the collaborative group improved more in some types of reasoning strategies than the other two conditions [91], both across the training and when comparing between pre- and post-test accuracy.

#### 3.1.6. Virtual week task

The Virtual Week (VW) task [106] is a board game containing 122 squares that represent times of day between 07.00 and 22.00, with one rotation of the board representing a single day. Participants roll dice to determine how far they move through the day, with some squares requiring drawing an ‘event card’. The card describes a common and realistic activity with three options (for example, going to see a movie with a friend, and choosing between romance, comedy, and adventure). Each of the options has a specific dice roll that it requires of the participant. Rolling the dice, moving across the board, and making decisions concerning the activities on the event cards are the main aspects of the VW task. In the study by Margrett *et al*. [90] participants had to perform 10 tasks (i.e. tell the experimenter of the tasks they would have to perform if done in real life), some of which recurred each time the VW task was played, and other tasks were specific to that trial. The participants were later asked what those tasks were and assessed on their accuracy. Margrett *et al*. [90] found that middle-aged adults (aged between 42 and 63 years) outperformed the older adults (aged between 67 and 82 years) as they were more accurate in remembering their performed tasks. In addition, the pairs of participants in the study actively worked together, most often tutoring each other while taking part in the task, which had a significantly positive effect on their performance.

#### 3.1.7. Black Box Task

The Black Box Task [107] has four atoms hidden in a 9 by 9 matrix. Each atom has an invisible field of influence, where light rays are reflected at a 90° angle. Participants must determine whether the atoms are on the grid, using the light rays that were shot into the matrix and their knowledge of whether the light rays are or are not deflected by the atoms. On the grid, only the exit and entrance points of the light rays are shown, not the path of the light itself, and they must be remembered by participants as they disappear once the participant indicates where an atom is hidden, or another light ray is added to the grid. Participants are given a booklet with instructions as well as practice trials before taking part in the task. Performance on the Black Box Task is measured through the number of correctly completed trials. In the study by Peter-Wight & Martin [53], older adults completed the task both together and individually. The results indicated that older adults performed better (i.e. completed more trials correctly) when they did the task together versus when they did the task individually, suggesting that collaborating on this task was more beneficial for older adults than completing the task alone.

#### 3.1.8. Bingo nutrition and health game

Seah *et al*. [79] used a task involving the traditional game of bingo paired with a learning component to study collaborative learning among older adults. The task was computer-based and provided participants with a 5 × 5 bingo sheet on the screen, as well as participants' overall scores. In the study, participants took part in groups of four to eight people, over the course of six weeks. They played two trials per session, and the task increased in difficulty each week. In line with bingo, a number would be called, and the participants would check their sheet. If the number was present on their sheet, the participant would select the number and would see a pop-up question about health. The questions varied in difficulty. If the participant answered the question correctly, the game would register the number on their sheet. To win, participants had to complete a row of numbers in any direction. Points were also awarded to the player based on question difficulty, and the player with the most points when ‘bingo’ is called was declared the winner of the game. The level of difficulty of the game can be adjusted by participants, with the options of easy, medium and difficult, corresponding to the difficulty of the pop-up questions. Seah *et al*. [79] found that older adults significantly improved their knowledge of health and nutrition, they felt more socially connected and had a more positive attitude towards digital games after taking part. The qualitative analysis of their interviews indicated that the theme of making new friends was particularly prevalent, and other themes that highlight the social aspect of the task and the gained knowledge of technology, health and nutrition.

#### 3.1.9. Everyday problem-solving task

The everyday problem-solving task used by Cheng & Strough [84] was a trip-planning task derived from previous research on planning and problem solving [108], with the decision to use planning a trip as it is a common experience for many adults. Participants were given an 11 × 17-inch laminated copy of the western half of the ‘United States Driving Distance Chart’ to plan the most efficient two-week round-trip route between Denver, Colorado and San Francisco, California. The route would be intended for someone else, with the purpose of attending a wedding. As well as being the most efficient, the trip had certain requirements; the trip would also have to include Salt Lake City, Las Vegas and Mount Rushmore, as well as one of two national parks (either Yellowstone National Park or Yosemite National Park). Additional restrictions were included, such as specific dates for events or visits, maximum hours of travel, and opening times. As the task requires consideration of multiple requirements and restrictions, participants were told that they were allowed to make minor errors to meet larger requirements (such as the wedding). Task performance was measured by route efficiency, overall completion accuracy (i.e. combination of the scores on all elements), component completion accuracy (e.g. city requirement completion, including visiting Las Vegas), the number of requirements completed (e.g. city visits, national parks, attending the wedding), route efficiency, and several types of errors. Cheng & Strough [84] found that collaborators made fewer errors and were overall more accurate than individuals. Older adults performed equally well or less well than younger adults on different elements of the task: the age groups were similar in route efficiency and planning errors, but older adults were overall less accurate, did fewer of the city and Mount Rushmore requirements of the task and were slower in completing the task.

#### 3.1.10. Wii Sports Resort

Lastly, Zhang *et al.* [99] used the Wii Sports Resort videogame to examine how older and younger adults learned new skills collaboratively over the course of six weeks. Wii Sports Resort is a videogame designed for the Nintendo Wii game console, in which players can take part in a variety of simulated sports (such as basketball and golf). In the study, a younger and older adult participant (multi-generational pairs) played the Wii Sports Resort cycling and canoeing games side-by-side for two weeks, and again in the last two weeks of the study after gaining some experience with the game. None of the participants had played those specific games before, though younger adults were more experienced overall as they reported playing similar videogames at least once a week. As such, the younger adults were intended to be the experts to help facilitate learning for the older adult participants. Through conversation analysis, results suggest that older adults learned through asking questions and receiving feedback from their younger partners, such as them pointing out errors made, orienting their attention to the key aspects of the game, or explaining concepts of the videogame and the console to their older adult partners.

### 3.2. Learning collaboratively versus alone

Surprisingly, out of the 29 studies examining collaborative learning, only seven compared learning together to learning individually (*n* = 7). Of these seven studies, five studies used a multi-week skills-based training course, most of which were to improve safe Internet usage skills [91,93,94,103,104]. For example, Xie [103,104] and Vazquez *et al*. [94] conducted multi-week training sessions on searching for health information online, using a tutorial designed by the NIH. All three studies found that there was no difference in study outcomes between those working alone and working together. These findings are in line with those of Margrett & Willis [91] and Saczynski *et al*. [93], who conducted multi-week training courses in inductive reasoning, who found that receiving training itself was helpful, and that performance improved independent of people learning alone or together.

The remaining two works used specific tasks to study individual versus collaborative learning in a single session. Cheng & Strough [84] used an everyday problem-solving task in which participants needed to plan a route with multiple requirements and restrictions. They found that pairs were more successful in incorporating the requirements of the trip, made fewer planning errors and had better overall performance on the task than individuals, though they did not outperform individuals on all aspects of the task (e.g. route efficiency, completed requirements, and number of calculation errors). Lastly, Peter-Wight & Martin [53] used the Black Box paradigm, a game in which participants must determine the location of 4 hidden atoms on a grid based on light rays that were shot into the grid. In this study, older adult pairs performed better at locating the atoms than individuals did.

These studies provide mixed evidence on whether learning together is more beneficial than learning alone. They suggest that collaboration offers benefits when using novel rule-based tasks that are completed in a single session, but less so when collaborating during longer training-based paradigms. In the latter case, it appears that the training itself is beneficial, independent of collaboration. In addition, the small number of studies comparing these two conditions also means there is currently a limited evidence base for determining whether and when collaborative learning is beneficial for older people, as it is difficult to assess the benefits of collaboration when not taking older adults' individual performance into account.

### 3.3. Familiar versus unfamiliar pairs

Of the 29 studies, six investigated collaborative learning with both familiar and unfamiliar pairs (*n* = 6). The materials used in these studies varied, with the most common being the referential task/Barrier Task (*n* = 3) or an IT training program (*n* = 2). None of the studies found a significant effect of familiarity (for older adults), suggesting that older adults’ performance was not affected by who they learned with. For example, Rodrigues *et al*. [92] used a referential task to study whether there is a difference in learning with a familiar and unfamiliar partner. Participants were instructed to complete as many trials as possible within 15 min. Results showed that older adults were relatively more efficient in communicating with an unfamiliar partner than when they collaborated with a familiar partner, as they used fewer words to describe the images. Also using a referential task, both Crompton *et al*. [86] and Gould *et al*. [88] did not find an effect of familiarity, as participants' learning performance (i.e. number of words and turns, as well as time to complete each trial) was the same across both the familiar and unfamiliar conditions. This trend persisted across different tasks. Using the Map Task, in which participants had to work together to complete a route of which each person only had half available to them, Crompton [82] found that participants’ learning performance (i.e. accuracy of the route, the number of words and turns, and time to complete the trial) did not differ when working with a familiar or unfamiliar partner.

Lastly, two studies by Xie [104,105] used training tutorials on searching for and evaluating online health information to assess the effect of familiarity. The findings are in line with that of other works investigating familiarity, as older adults improved to a similar extent independent of who they worked with.

In summary, relatively few studies have investigated whether collaborative learning differs between familiar and unfamiliar pairings. However, across these six studies, different designs were used, and studies obtained a similar outcome regarding the (lack of an) effect of familiarity on older adults' learning. These findings suggest that the benefit of learning collaboratively in older age does not depend on a pre-existing relationship between partners.

### 3.4. Younger versus older adults

Studies examining younger versus older adults are more common than other research categories within collaborative research in older age, with 10 of 29 studies including a younger adult group (*n* = 10). The majority of these 10 studies used the referential task/Barrier Task as their learning material. The outcomes in terms of age differences using this specific material vary; 4 of the 7 studies using a referential task found that younger adults outperform older adults in terms of accuracy, speed or using optimal descriptions [87–89,98]. However, three of the seven studies using referential tasks reported that older adults’ performance on the referential task (i.e. words spoken, turns taken, time to complete, number of trials completed) was initially poorer but caught up with younger adults over time [7,86,92]. These three studies that found no later age differences incorporated more trials than the other studies using a referential task. For example, Crompton *et al*. [86] included 9 trials of 12 tangrams per condition, Derksen *et al*. [7] had 6 trials per condition and 24 trials overall, and Rodrigues *et al*. [92] asked participants to complete as many trials as possible within 15 min.

In addition, while the referential task's design is similar throughout these studies, there are some differences in its execution or design; for example, most studies use tangrams but Yoon & Stine-Morrow [98] instead used common yet ambiguous images instead. They also used a computerized version of the task. Yoon & Stine-Morrow [98] found that younger adults outperformed older adults on the task. However, in their study there is the potential of additional difficulty for older adults as the task used more complex visual stimuli and required the use of computers. Older adults have been found to have less knowledge of technology than younger adults [96], and equally report higher levels of discomfort with technology [79]. As such, it might be that age differences on the referential task depend on the number of task trials and its execution (i.e. in person or computerized), as this may explain the variety of findings in studies using this paradigm.

The other three studies examining age differences used three different tasks: an everyday problem-solving task (involving planning a trip), an adapted version of the VW task, and an adapted version of the Map Task. The first two studies found that younger adults performed better than older adults [84,90], whilst the latter found that there was no difference between age groups in their performance on the Map Task [82].

### 3.5. Qualitative works

As most included works on collaborative learning use quantitative methods, this section will specifically discuss studies using solely qualitative methods. Three of the included works (*n* = 3) use qualitative methods such as semi-structured interviews to examine how working together affects older adults' learning. Zhang *et al*. [99] observed how mixed-age pairs of older and younger adults worked together when playing a Wii Sport videogame and discussed how older adults learned to play the game. Through conversations with their younger counterparts, who were generally more skilled in playing videogames, older adults learned through asking questions and benefited from younger adults’ feedback, such as pointing out tasks or elements to be completed, as well as correcting mistakes.

Sayago *et al*. [78] conducted multiple sessions on learning computing skills, in which participants were encouraged to collaborate, but not required to do so. Participants discussed how they especially enjoyed the social aspect of the sessions, and how they reflected and learned through conversations during and after sessions. However, once more comfortable with their skills, some purposely sought out new partners to learn with during sessions. The conversations between participants often also involved forms of cueing and supporting the other person in their recall of information.

Lastly, Wei *et al*. [97] interviewed romantic couples on their Internet usage, using semi-structured and open-ended questions. Participants reported that one partner was often more proficient at using the Internet and would support the learning process of their less knowledgeable partner.

Overall, the findings of these three studies suggest that older adults consider working together to be useful, as it is easier to obtain knowledge [78,97,99], enjoy the social aspects of collaborating [78,97], and some that many prefer working with someone they know [78].

### 3.6. Computer versus human collaboration

Of the works included, two studies (*n* = 2) examined whether collaboration in older adults differed when working with a (perceived) human partner or with a computer. In these studies [82,85], participants were told that they would be working with a human partner and would communicate through a computer, as well as working with a computer program, to complete the task. In fact, feedback in both conditions was provided by a computer, but the perceived human partner's programming used natural language. Two different tasks were used in the studies: the adjusted Map Task [82] and a referential task/Barrier Task [85]. In the study using the Map Task, participants engaged less with the computer partner, recalled less of that session, and reported finding it more difficult compared to learning with their perceived human partner. In the study using the Barrier Task, participants in the computer condition were slower, took fewer turns, and recalled less. This finding suggests that participants were less involved with the computer partner, as they took longer but also engaged less to acquire feedback. Overall, findings indicate that older adults prefer working with a perceived human partner compared to a computer, even if this perceived human partner is also a computer that uses natural speech. This supports the argument that collaborative learning offers social benefits.

### 3.7. Familiar-only or unfamiliar-only

This section discusses works that have used unfamiliar or familiar participants only, and did not compare different learning conditions or groups, such as younger versus older, or familiar versus unfamiliar.

#### 3.7.1. Familiar-only

Most of the works looking at a single group of collaborating pairs involved familiar pairs, such as spouses or friends. Four studies (*n* = 4) include familiar-only pairs to investigate collaborative learning. In the two papers by Vrkljan [95,96] older married couples had to program a GPS device across several trials. The couples who had more experience with technology were able to complete tasks faster, with fewer errors, and provided less assistance to each other [96], while those with less technology experience offered mostly support through encouragement to one another [95]. The study highlights how prior experience with technology was related to the type of collaboration and support partners offered each other. Wood *et al*. [80] conducted several training sessions on developing computing skills with older adults who were largely familiar with one another as they lived in the same assisted living facility. The results showed that participants were overall engaged with their training partner, as there was an increase in positive collaborative behaviors (i.e. asking the partner, sharing the mouse, turn-taking with partner). Of the participants taking part, more than half found working as a pair useful (56.1%) and effective (59.1%), and a little under half of participants indicated that their partners had contributed to their learning (48.5%) [80].

Lastly, in Wei *et al*. [97], spouses were interviewed about their Internet usage. The main reasons behind older adults using the Internet collaboratively are related to day-to-day tasks (such as grocery shops), curiosity (such as solving problems or answering questions) and sharing the experience of searching online together, all part of relationship maintenance. This suggests that familiar pairs take the other person's interests, preferences and needs into account, as well as assigning roles to each other that fit each person's skill set or knowledge.

#### 3.7.2. Unfamiliar-only

Four studies used unfamiliar-only designs (*n* = 4), in which participants learned together with a stranger. Two of these studies are by Morrow *et al*. [83], in which pairs of participants completed four medication scheduling tasks, each with differing medication and patient complexity. The study included three conditions: a medication scheduler, a blank piece of paper to write on, and no aid. The first study showed that using any aid was associated with higher accuracy, especially when the medical scenario was complex. However, participants completed the problems more slowly when using the medication scheduler.

In the second study by Morrow *et al*. [83], the no-aid condition was removed, participants were given more practice with their aid beforehand, and a measure of delayed recall was included. Participants' schedules were more accurate and were completed faster when participants used the medication scheduling aid. The findings of both studies suggest that a medication scheduling aid is a helpful tool when collaboratively creating a medication schedule, more so than a blank piece of paper or no aid, especially when the problem is complex. However, participants’ long-term memory of the information is low, likely due to cognitive load of the task in question.

Using the MedTable in Morrow *et al*. [83], Kannampallil *et al*. [81] further explored the effect of aids on medication scheduling. Participants were given either the existing MedTable, an electronic version of the MedTable named e-MedTable, or a common yet unstructured aid named MedCard. Participants were randomly paired with an unfamiliar partner and asked to create four medication schedules together based on medical scenarios. The four medical scenarios were either simple or complex. Results showed that older adults who used the structured medication schedulers, the paper-based MedTable and eletronic e-MedTable, created more accurate schedules and participants found the structured aids easier to use than the unstructured MedCard. However, unlike in Morrow *et al*. [83], in which the MedTable improved medication scheduling for complex medical scenarios, the results of Kannampallil *et al*. [81] did not show the same finding. This might be due to the differences between conditions in the studies; in the two studies by Morrow *et al*. [83], participants were given the MedTable, a blank sheet of paper or no aid at all, while Kannampallil *et al*. [81] compared between three types of medication scheduler across conditions. As such, it may be that the added benefit of using the MedTable compared to little or no structured aid does not appear when solely comparing between medication scheduling aids.

Lastly, Sayago *et al*. [78] interviewed older adults who attended classes on computer skills at adult educational centres. Participants reported that they found it more difficult to work with strangers compared to participants they had worked with before during earlier sessions, as they either had different strategies or would have to create one together. As such, most participants chose to learn with the same partner during their initial sessions.

Overall, studies that chose to use only familiar or unfamiliar participants did so as it was essential to their research questions, such as how married couples used the Internet together, or these group compositions were samples of chance, as people were included due to the activities they took part in or their location (such as nursing homes or the library). These works contribute to the research area through an increased focus on understanding participants' arguments for and against collaborative learning, their experience of doing a specific learning task or course together, and whether prior experience or skill impact collaborative learning in older age.

## 4. Discussion

As Western populations are ageing, age-related cognitive decline will affect more people than previously. Concomitantly, people are frequently working until later in life before retiring. As such, negating age-related effects on cognition is highly timely and essential, as is understanding how learning new knowledge and skills in older age can be optimized. This systematic review investigated whether collaborative learning benefits older adults, and under what circumstances learning with another person is most beneficial. We screened the titles and abstracts of 6629 works, assessed the full text of 77 works, and found 27 works that met all inclusion criteria. Of these 27 works, two works included multiple studies. As such, a total of 29 studies were included in the review. Across the 29 studies, there was a wide range of materials and study designs, as well as a focus on a variety of different questions (though the use of a referential task, such as the Barrier Task, was most common). Because of this, the studies included are separated into categories, both in the results section and in this discussion section.

Whether older adults learn better collaboratively or individually is arguably the most significant question in this area of research. Of the 29 studies, only seven looked at the effectiveness of learning together while including an individual learning condition. Overall, these studies report mixed findings, with some finding that collaborative pairs were more successful than individuals, while other studies report no differences between conditions. However, collaborating with a partner seems most beneficial in studies that used novel rule-based tasks, while longer-duration training programmes resulted in similar outcomes for those working alone and working together. One possible explanation for this is that collaborating is most helpful when learning novel tasks, as these offer the most potential for cross-cueing between learners as well as collaboration to recall the rules or details of the task (as seen in [99]). By contrast, longer and more structured programmes may offer enough opportunities for individuals to acquire all the information by themselves. In addition, many of the skill training sessions included instructors or facilitators who were available to participants, meaning that information could still be obtained from another person even when working alone.

In general, however, this review highlights that there is limited evidence for how, why, and when collaboration provides benefits compared to individual learning, and this is an important target for future research. For example, it is worth exploring whether someone is a collaborator or merely a facilitator in the learning experience (as even the inclusion of some minor social interaction during the learning experience may be beneficial), and how this differs across types of learning (e.g. novel tasks, training programmes). In practical terms, many studies currently do not include a baseline of a person's learning capability due to the lack of an individual condition. Future work would be advised to include an individual condition when investigating collaborative learning, with individual learning materials as close as possible to those of the collaborative learning condition.

Several studies also looked at whether the potential benefit of working together depends on familiarity between learning partners. The six studies that investigating this found similar outcomes, despite differences in design, namely the lack of an effect of familiarity on older adults' learning. This suggests that benefits of learning together at an older age do not depend on a pre-existing relationship between partners and may instead be due to the social component of working with another person or the potential for cross-cueing of information. This is further supported by works that involve learning with a computer instead of a human partner, such as Crompton & MacPherson [85] and Crompton [82]. In these two studies, participants’ performance worsened when they believed they were working with a computer, and they reported finding it more difficult working with the computer compared to the perceived human partner (despite both conditions being entirely computerized). It is important to note that, while there were no quantitative differences in learning success, in some studies older adults reported finding it more difficult to work with an unfamiliar partner. This was the case in a study that offered a flexible learning environment in which participants were allowed to choose their partner [78] (unlike most studies which assign participants to conditions randomly). It may be that older adults prefer learning with a familiar person over a stranger when they are able to choose their partner. In addition, older adults reported that they enjoyed working with another person, actively sought out others to work with, and that learning together served a social purpose for them. This finding is not specific to collaborative learning; research on collaborative recall also reports mixed findings on whether existing relationships benefit collaborative recall [55], while older adults also report a preference for working with a familiar person such as their spouse [51].

The lack of a familiarity effect may be helpful when designing learning programmes for older adults, as it indicates that a successful learning experience can be achieved with an unfamiliar collaborator. Thus, older people do not need to attend learning sessions with a known learning partner, which is especially useful for older adults who live alone or experience loneliness.

Studies that examine learning in younger versus older adults were the most common type of study in this review, with almost half of studies including a younger adult sample (ten out of 29 studies). In addition, this category is the most uniform in terms of the materials that were used: seven of the ten studies used a referential task to compare age groups. However, there were differences in how these referential tasks were executed, notably the length of the task. The three studies that found no age differences included more trials than the four studies that reported age differences in favour of younger adults. More specifically, the findings of Derksen *et al*. [7] and Crompton *et al*. [86] indicate that it is possible that the difference in performance between younger and older adult pairs is present when commencing a novel task but decreases over time. The lack of age differences in some of these studies suggests that older people can sometimes perform novel tasks as effectively as young people, when working in collaborative settings. Thus, one intriguing possibility is that collaboration can ameliorate age-related declines in learning. Confirming this requires more studies that directly compare learning alone versus collaboratively across age groups, to see if older people show a larger collaborative benefit than younger adults. Only one of the studies we reviewed [84] used such a 2 × 2 (age × collaboration) design. In addition, it is currently unclear how many trials or how much time is required for age differences to disappear on tasks such as the Barrier Task, as studies differ in their design. For example, Crompton *et al*. [86] included 9 trials per condition, and participants took part in two conditions. Derksen *et al*. [7] had 24 total trials per participants, with 6 trials conducted in each of 4 sessions, with 2 sessions per day. Despite both using a larger number of trials, the studies differ in their design, making a direct comparison difficult. As such, future work may want to examine how quickly age differences disappear, on the Barrier Task as well as other learning tasks.

### 4.1. Recommendations for future research

The included works each contribute unique designs and results to the understanding of collaborative learning in older age. However, there are still gaps remaining in the literature that could be addressed. Firstly, of the studies on collaborative learning in older age in this systematic review, only seven had an alone versus collaborative design. To fully understand how and when older people benefit from working with another person, a baseline of individual learning performance is important. As such, this is an important consideration for future research on this topic. Secondly, almost all studies used learning pairs of similar ages. As such, it would be valuable to investigate how the effects of collaboration change (or remain the same) when working with a partner from a different age group. Collaboration between intergenerational pairs may further benefit older adults, particularly when learning to use new technology, as older people can benefit from the speed or knowledge of their younger partner (as seen in [99]). Thirdly, to our knowledge, no existing research assesses the long-term benefits of collaborative learning for older adults beyond several weeks after the initial learning experience. Future research could include the adoption of a longitudinal design to assess whether collaborative learning leads to long-term learning gains compared to learning alone, or whether there are age differences in the long-term gains of learning together (as some studies found that age differences disappeared when including a larger number of trials). Lastly, the qualitative studies in this review offer insight into the experience of older adults and how learning and conversation between partners occur. This review found both qualitative and quantitative studies that investigate this topic, yet there are few that use a combined mixed methods approach to assess the benefits of learning together in older age (such as [80]). Mixed methods could offer insights beyond quantifiable learning performance or observed behaviors. By combining the strengths of both quantitative and qualitative research, we may gain a more rounded and nuanced understanding of how collaborative learning occurs and its benefits.

## Data Availability

Materials related to this paper, including screening manual, logbook, and references of imported works, have been uploaded to the Open Science Framework: https://osf.io/8xvqf/. The data are provided in electronic supplementary material [109].

## Footnotes

1 We initially included PsychInfo in our literature search but the works resulting from the search were highly irrelevant. As such, we removed PsychInfo as one of the databases with editorial approval granted on 1 October 2022.

2 We have chosen to use Google Scholar as a supplementary search engine and to include only the first 5 pages of results, in line with prior work on how best to utilize Google Scholar for systematic reviews [74–76].

3 Both Prospero and EThOS showed no results when using the full list of keywords used for other databases, and EThOS does not accept asterisks or the root of words (i.e. collaborat* or collaborat instead of collaborative). In the case of EThOS, this resulted in some key results being omitted. As such, both databases were searched using a broader term that yields a higher number of results. This change was approved by the editor on 14 March 2022.
